# Disrupted Slit-Robo signalling results in membranous ventricular septum defects and bicuspid aortic valves

**DOI:** 10.1093/cvr/cvv040

**Published:** 2015-02-17

**Authors:** Mathilda T.M. Mommersteeg, Mason L. Yeh, John G. Parnavelas, William D. Andrews

**Affiliations:** Department of Cell and Developmental Biology, University College London, 21 University Street, London WC1E 6DE, UK

**Keywords:** Heart development, Slit-Robo signalling pathway, Notch signalling pathway, Membranous ventricular septum defect, Bicuspid aortic valves

## Abstract

**Aims:**

The mesenchymal cushions lining the early embryonic heart undergo complex remodelling to form the membranous ventricular septum as well as the atrioventricular and semilunar valves in later life. Disruption of this process underlies the most common congenital heart defects. Here, we identified a novel role for Slit-Robo signalling in the development of the murine membranous ventricular septum and cardiac valves.

**Methods and results:**

Expression of Robo1 and Robo2 receptors and their ligands, Slit2 and Slit3, was present in or adjacent to all cardiac cushions/valves. Loss of *Robo1* or both *Robo1* and *Robo2* resulted in membranous ventricular septum defects at birth, a defect also found in *Slit3*, but not in *Slit2* mutants. Additionally, *Robo1;Robo2* double mutants showed thickened immature semilunar and atrioventricular valves as well as highly penetrant bicuspid aortic valves. *Slit2* mutants recapitulated the semilunar phenotype, whereas *Slit3* mutants displayed thickened atrioventricular valves. Bicuspid aortic cushions were already observed at E12.5 in the *Robo1;Robo2* double mutants. Expression of *Notch-* and downstream *Hey* and *Hes* genes was down-regulated in *Robo1* mutants, suggesting that reduced Notch signalling in mice lacking *Robo* might underlie the defects. Luciferase assays confirmed regulation of Notch signalling by Robo.

**Conclusion:**

Cardiac defects in mutants for Robo or Slit range from membranous ventricular septum defects to bicuspid aortic valves. These ligands and receptors have unique functions during development of specific cardiac cushion derivatives, and the Slit-Robo signalling pathway likely enforces its role by regulating Notch signalling, making these mutants a valuable new model to study cardiac valve formation.

## 1. Introduction

Cardiac septation and valve formation are complex processes requiring precise gene regulation, and even the smallest disruption can lead to congenital defects such as ventricular septum defects or malformed and malfunctioning valves. The membranous interventricular septum and the atrioventricular and semilunar valves develop from primitive jelly-like cushions lining the early myocardial heart tube. The endothelial cells covering the cushions invade the underlying matrix by epithelial-to-mesenchymal transformation, where they proliferate and reshape the primitive cushions into relatively thick valve-like cellular structures by mid-gestation. Subsequently, the cushions remodel to form the thin mature leaflets of the atrioventricular and semilunar valves and the upper membranous part of the ventricular septum.^1–3^

In addition to the epithelial-to-mesenchymal transformation, parts of the cardiac cushions, including the future membranous ventricular septum, receive contributions from neural crest cells invading the arterial pole of the heart, adding to the complexity of the region.^1^ Correct formation and alignment of the cushions requires accurate signalling from within as well as from neighbouring tissues such as the myocardium, endocardium, second heart field, and neural crest. Major signalling pathways, such as Notch, Bmp, and Wnt, are crucial for cushion development and, in turn, regulate and are regulated by a plethora of transcription factors such as the Tbox and Sox families.^1,2–5^

Here, we describe a new candidate involved in membranous ventricular septum and valve formation, the Slit-Robo signalling pathway. The Roundabout (Robo) transmembrane receptors and their Slit ligands, initially identified in *Drosophila*,^6,7^ became known for their roles in axonal guidance in the embryonic nervous system. However, since this discovery, many new roles for the Slit-Robo signalling pathway have been identified, mainly in cancer and embryo development.^8,9^ In *Drosophila* and zebrafish, Slit-Robo signalling plays key roles in cell adhesion during cardiac cell polarization, morphogenesis, migration, and lumen formation.^10–13^ During murine heart development, roles for Slit-Robo signalling have now been described in cardiac chamber formation^14^ and cardiac neural crest migration and adhesion.^15,16^ Disrupted signalling results in partial absence of the pericardium and abnormal venous connections to the heart.^16^ However, knowledge of the pathway during mammalian heart development is limited. Its identified functions during neural crest migration and adhesion combined with the known expression patterns of *Slit/Robo* in the neural crest, outflow tract, and atrioventricular cushions^14–16^ suggest an additional role for this signalling pathway in the formation of these areas.

We have now identified a broad spectrum of cardiac defects in mutants for *Robo* and *Slit*, ranging from membranous ventricular septum defects to bicuspid aortic valves. We show that these ligands and receptors have unique functions during the development of the different cardiac cushion derivatives and that the Slit-Robo signalling pathway likely enforces its role by regulating Notch signalling. This is the first study indicating the involvement of this pathway in the development of membranous ventricular septum defects and bicuspid aortic valve disease, and the high penetrance of bicuspid aortic valves can make the *Robo1/2* double mutant a valuable new tool to study the aetiology of this common human congenital disorder.

## 2. Methods

Transgenic mice and experimental procedures for *in situ* hybridization, immunohistochemistry, cell counts, three-dimensional reconstruction, volume and length measurements, qPCR, luciferase assays, and statistical analyses are provided in the Supplementary material online. All experimental procedures were performed in accordance with the UK Animals (Scientific Procedures) Act 1986 and institutional guidelines.

## 3. Results

### 3.1 Slit and Robo expression in the outflow tract and atrioventricular region

**Figure 1:**
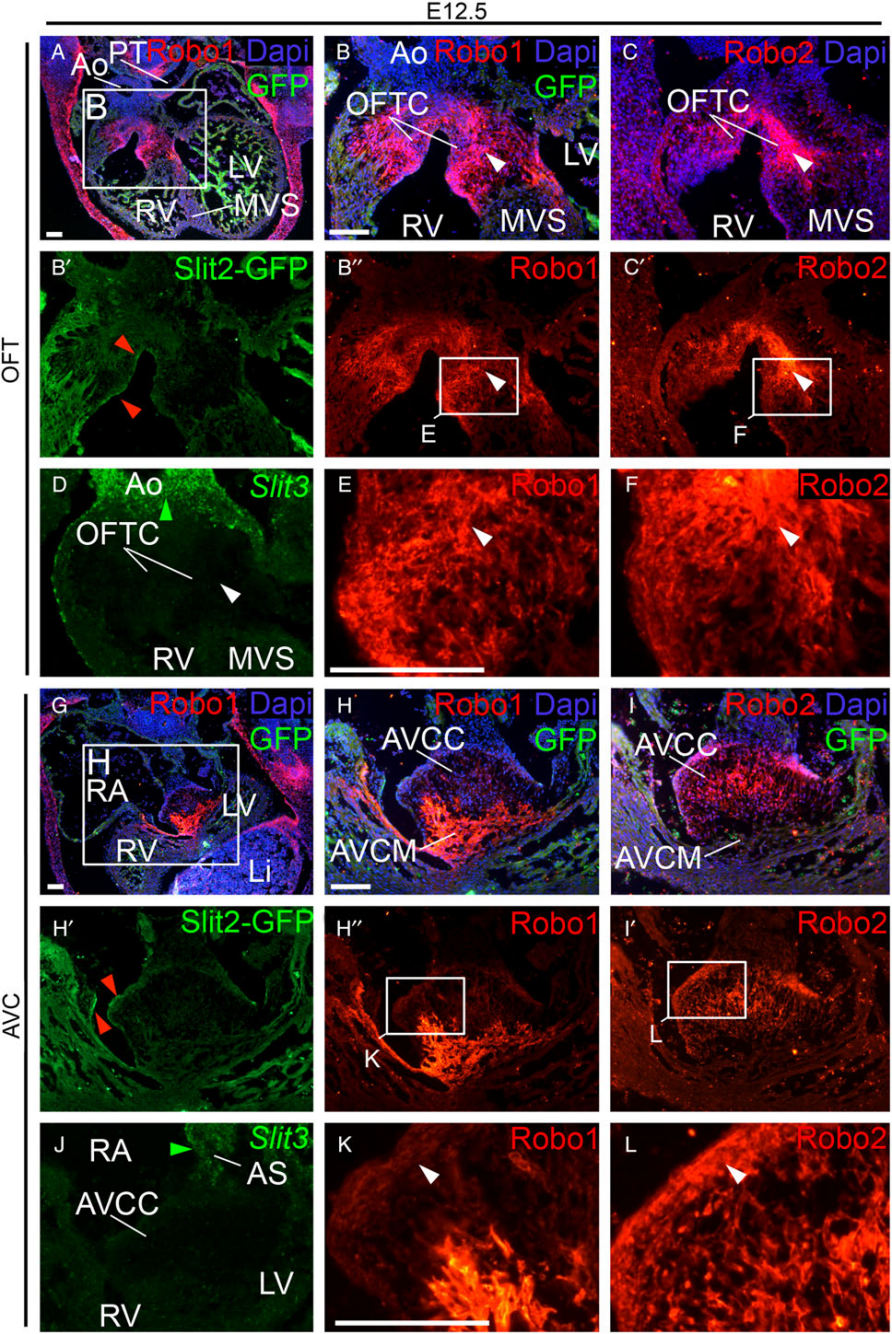
Slit and Robo expression patterns in and surrounding the cardiac cushions. (*A*–*L*) immunohistochemistry (Robo1, Robo2, and Slit2-GFP), DAPI, and *in situ* hybridization (*Slit3*) staining at E12.5 in the outflow tract and atrioventricular cushion regions. Red arrowheads indicate the expression of Slit2 in the endocardium lining the cushions. (*E*, *F*, *K*, and *L*) show details of Robo1 and 2 expression in the indicated cushion regions. White arrowheads point to the region where both Robo1 and Robo2 are expressed. (*D* and *J*) Green arrowheads point to Slit3 expression in the outflow tract vessels and atrial septum, while Slit3 is not detectable in the cushions. Per stage for all genes analysed, *n* ≥ 3 embryos. Ao, Aorta; AVC, atrioventricular canal; AVCC, atrioventricular cushion; AVCM, atrioventricular canal myocardium; GFP, green fluorescent protein; Li, liver; OFT, outflow tract; OFTC, outflow tract cushion; MVS, membranous ventricular septum; PT, pulmonary trunk; RA, right atrium; R/LV, right/left ventricle. Scale bars depict 100 µm.

We have previously reported the overall expression patterns of *Robo1*, *Robo2*, *Slit2* and *Slit3* in the murine heart and migrating cardiac neural crest.^16^ The presence of these genes in and surrounding the cardiac cushions prompted us to study their expression in these regions in more detail. As we have previously shown that *Robo3* is not expressed inside the heart and *Robo4* only in the coronary circulation, we excluded these receptors from our study. Robo1 was expressed in the outflow tract and atrioventricular cushions, and subsequently valves, as well as in the atrioventricular canal myocardium throughout embryonic development (*Figure 1A*, *B*, *E*, *G*, *H,* and *K*; see Supplementary material online, *Figure S1A–H*),^16^ the only exception being its disappearance from specifically the aortic semilunar valves just before birth (data not shown). Robo2 was never observed in the myocardium but was present in both the outflow tract and atrioventricular cushions, and later valves, all through development (*Figure 1C*, *F*, *I*, and *L*; see Supplementary material online, *Figure S1A–H*). In the outflow tract around E12.5, expression of Robo2 was highest in the cushion area contributing to the separation of the aortic and pulmonary outflow within the heart (*Figure 1C* and *F*; see Supplementary material online, *Figure S1C*).

*Slit1* was not expressed in the heart at any stage (see Supplementary material online, *Figure S1A* and data not shown) and was not analysed further. Besides its most evident expression in the ventricular trabecular myocardium, Slit2 was present in the endocardium lining both the Robo1- and Robo2-positive outflow tract and atrioventricular cushions and valves, and in the aortic semilunar valves just prior to birth (*Figure 1A*, *B*, *G,* and *H*; see Supplementary material online, *Figure S1A–H*). In contrast, Slit3 was not expressed in the cardiac cushions or valves (*Figure 1D* and *J*; see Supplementary material online, *Figure S1A–H*) but was found in the outflow tract and atrial myocardium adjacent to the cardiac cushions.

### 3.2 Absence of Slit-Robo signalling results in membranous ventricular septum defects

**Figure 2:**
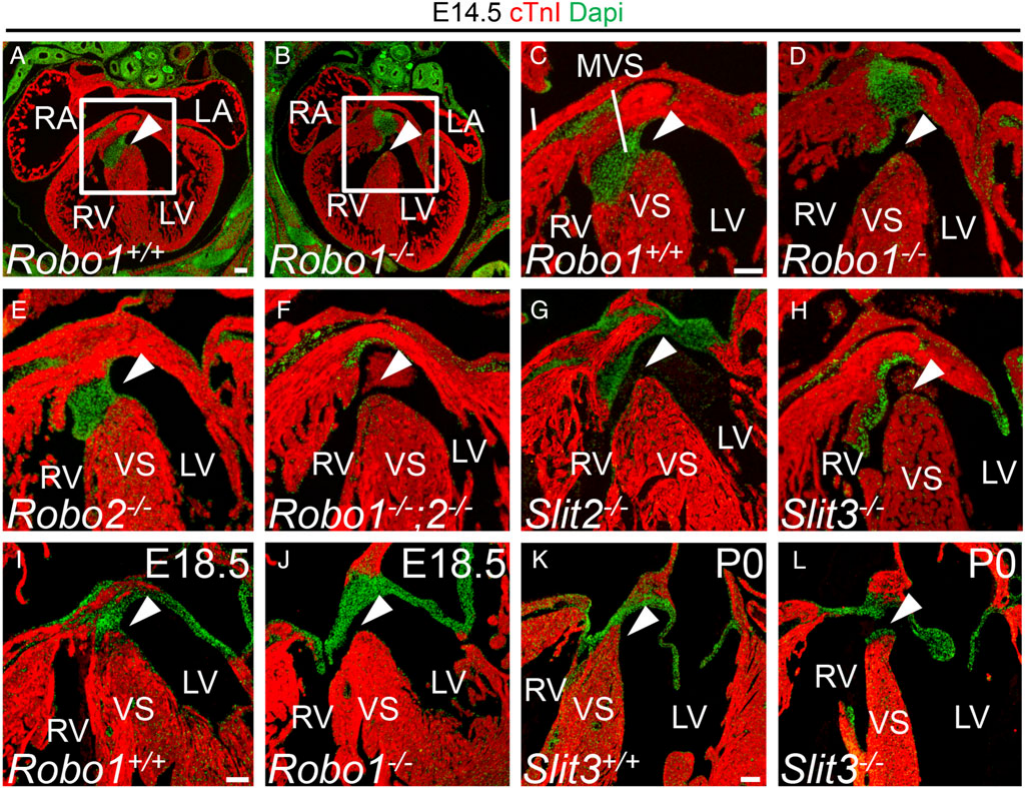
Disrupted Slit-Robo signalling results in membranous ventricular septum defects. (*A*–*L*) immunohistochemistry for cardiac Troponin I (cTnI) and DAPI on *Robo1^+/+^* (*A*, *C*, and *I*), *Robo1^−/−^* (*B*, *D*, and *J*), *Robo2^−/−^* (*E*), *Robo1^−/−^;Robo2^−/−^* (*F*), *Slit2^−/−^* (*G*), *Slit3^+/+^* (*K*) and *Slit3^−/−^* (*H* and *L*) hearts. The valves and the membranous ventricular septum are visible as green DAPI staining. White arrowhead points to the presence or absence of the membranous ventricular septum (see *Table 1* for numbers of embryos analysed). VS, (muscular) ventricular septum. For other abbreviations, see the legend of *Figure 1*. Scale bars depict 100 µm.

These expression patterns, combined with the identified roles of Slit-Robo during neural crest migration and adhesion, imply that defective signalling might result in cardiac valve and membranous septum defects. Therefore, we first analysed mice mutant for Robo1 and/or Robo2 receptor for septum defects. At E14.5, we noticed absence of the membranous ventricular septum combined with an overriding aorta in 60% of embryos lacking *Robo1* (*Figure 2A*–*D*; *Table 1*), whereas the ventricular septum was closed in all littermate controls at this stage. The membranous ventricular septum closes the communication between the right and left ventricles by fusion of the outflow tract cushions with the atrioventricular cushions and is normally completed at E14.5. *Robo2* mutant mice did not show any defects, whereas *Robo1;Robo2* double mutants showed a phenotype similar to *Robo1* lacking mice, indicating that Robo1 is the main Robo receptor required for the development of the region (*Figure 2E* and *F*; *Table 1*). All double mutants analysed exhibited a septum defect, suggesting an additional role for Robo2, although this difference might be caused by the lower numbers of double mutants analysed. The membranous septum defect was still present in 30% of *Robo1* mutants at E18.5 (*Figure 2I* and *J*; *Table 1*), suggesting in one-third of mutants closure is merely delayed as the Mendelian ratio at birth is normal.^17^

**Table 1:** The prevalence of membranous ventricular septum defects and bicuspid aortic valves in the absence of genes of the Slit-Robo signalling pathway

	Membranous ventricular septal defects				Bicuspid aortic valves	
	E14.5		E18.5		E18.5	
Embryo	+/+;+/−	−/−	+/+;+/−	−/−	+/+;+/−	−/−
*Robo1*	0/9	6/10	0/10	3/10	0/10	0/10
*Robo2*	0/5	0/5	0/6	0/8	0/6	0/8
*Robo1;Robo2*	0/3^a^	3/3^a^	na	na	0/3^a^	3/3^a^
*Slit2*	0/6	2/6	0/8	0/7	0/8	1/7
*Slit3*	0/5	2/5	0/4^b^	1/4^b^	0/4^b^	0/4^b^

The prevalence of membranous septum defects and bicuspid aortic valves in *Robo1*, *Robo2*, *Robo1;Robo2*, *Slit2*, and *Slit3* mutants at the indicated developmental stages.

^a^*Robo1;Robo2* was analysed at E15.5.

^b^*Slit3* was analysed at P0; na means not analysed.

We next analysed mice mutant for Slit2 and Slit3 ligands. Interestingly, both mutants showed membranous ventricular septum defects at E14.5 (*Figure 2G* and *H*). However, the prevalence was lower compared with *Robo1* mutants (*Table 1*). The septum defects in *Slit2* lacking animals were relatively small, whereas the *Slit3* knock-outs showed a phenotype more similar to *Robo1* mutants. Of the ligand mutants, only mice lacking *Slit3* showed a septum defect at P0 (*Figure 2K* and *L*; *Table 1*), indicating that Slit3-Robo1 interaction is most crucial for membranous ventricular septum development, but Slit2 and Robo2 are also involved.

**Figure 3:**
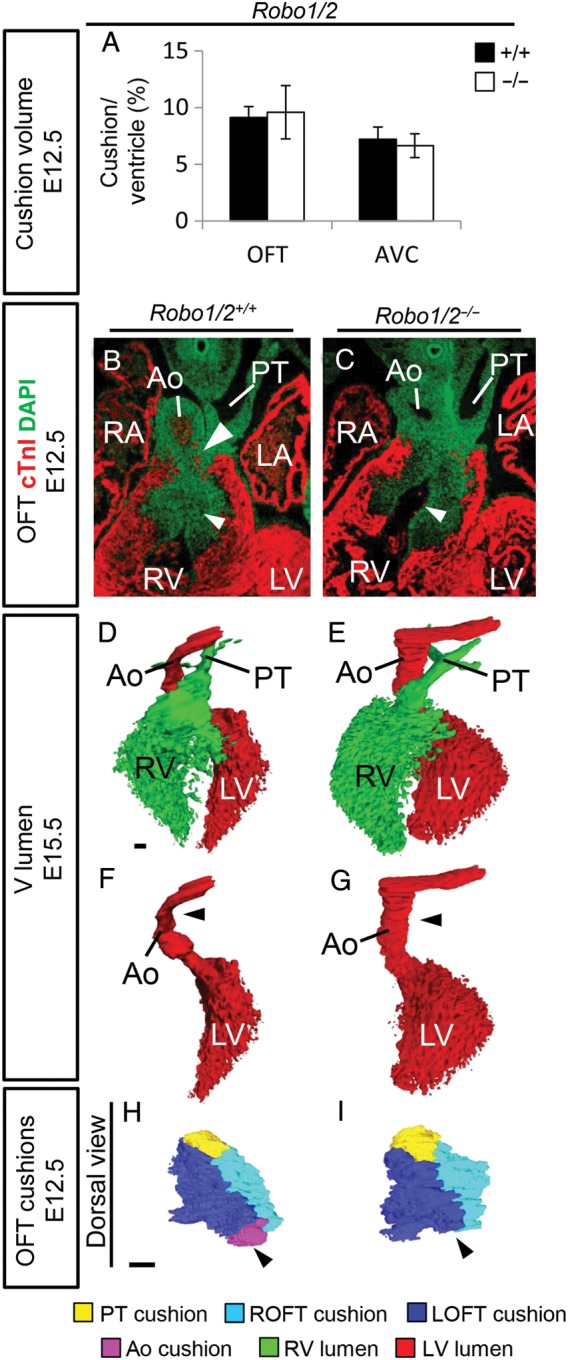
Defects in cushion formation in Robo1/2 double mutants. (*A*) Measurements of the OFT and AVC cushion volume corrected for ventricular volume (*n* = 3; OFT, *P* = 0.51; AVC, *P* = 0.27; Mann–Whitney *U* test). (*B*) Immunohistochemistry for cardiac Troponin I (cTnI) and DAPI on *Robo1^+/+^;Robo2^+/+^*, and *Robo1^−/−^;Robo2^−/−^* mutants. White arrowheads indicate increased space between OFT cushions in the mutant at E12.5. (*D*–*G*) Three-dimensional reconstructions of the ventricular lumen and outflow tract vessels of E15.5 *Robo1^+/+^;Robo2^+/+^* and *Robo1^−/−^;Robo2^−/^*^−^ hearts showing reduced rotation of the aorta in the mutant (black arrowheads), most clearly visible after removing the right ventricle and pulmonary trunk (*F–G*). (*H*–*I*) Three-dimensional reconstructions of the outflow tract cushions of E12.5 *Robo1^+/+^;Robo2^+/+^* and *Robo1^−/−^;Robo2^−/−^* embryos showing the absence of the aortic posterior cushion in the Robo1/2 mutant. *n* = 3. For abbreviations, see the legend of *Figure 1*. Scale bars depict 100 µm.

To identify the cause of the ventricular septum defects, we first analysed cushion development at earlier stages. At E12.5, we did not observe defects in the cushions of any of the mutant mice analysed (data not shown), except for *Robo1;Robo2* mutants. The size of the cushions was unaltered between the double mutants and wild-type littermates at E12.5 (*Figure 3A*). However, we observed reduced closure of outflow tract cushion regions that were already fused in wild-type littermates at this stage (*Figure 3B* and *C*; see Supplementary material online, *Figure S2A and B*). This region of impeded closure corresponded exactly to the outflow tract cushion region highly expressing both Robo1 and Robo2 (*Figure 1A*–*L*; see Supplementary material online, *Figure S1C*). As a result, the double mutants displayed a persistent connection between the right and left ventricle, while this connection was already closed in wild-type littermates at this stage (see Supplementary material online, *Figure S2C and D*).

Membranous ventricular septum defects can be caused by reduced contribution of the cardiac neural crest or second heart field to the outflow tract, resulting in aberrant alignment of the outflow tract vessels,^4,5^ or defects in endothelial-to-mesenchymal transformation and maturation of the cardiac cushions.^1,2^ Robo1, Robo2, and Slit3 are all expressed in the cardiac neural crest, while Slit2 is present in the neighbouring endoderm.^16^ As we previously demonstrated that increased apoptosis in the neural crest underlies the pericardial defects observed in Robo1 mutants,^16^ as well as the fact that the normally highly Robo1 and Robo2-expressing outflow tract cushion region showing reduced closure in the double mutant is neural crest derived (see Supplementary material online, *Figure S3A*), we started with analysing neural crest contribution to the outflow tract cushions in *Robo1;Wnt1^cre^;R26R^EYFP^* embryos. We did not observe increased apoptosis over the different time points between E10.5 and E12.5, and correspondingly neural crest contribution to the outflow tract was not significantly reduced at E11.5 (see Supplementary material online, *Figure S3B–D*). Also, no increase in apoptosis was observed in *Slit3* mutants (E12.5; see Supplementary material online, *Figure S3E*). These results do not completely rule out involvement of the neural crest in the defective formation of this region, but at least suggest additional mechanisms, likely second heart field defects, as *Robo1* as well as *Slit2* and *3* are expressed in the *Isl1*-positive second heart field at E10.5 (see Supplementary material online, *Figure S4A*). As both second heart field and neural crest defects can cause alignment defects of the outflow tract vessels, resulting in septum defects, we next analysed the arterioventricular alignment in *Robo1* mutants at E14.5. The aorta and pulmonary trunk were normally separated in all mutants (see Supplementary material online, *Figure S4B–D*; data not shown). Although we did not observe any major defects, we noticed a difference in outflow tract vessel alignment, with the aorta slightly more to the right of the pulmonary trunk (see Supplementary material online, *Figure S4B and C*). Three-dimensional analysis of *Robo1* mutant hearts further indicated reduced rotation of the outflow tract vessels and mainly the aorta (black arrowheads; see Supplementary material online, *Figure S4D*). This seemed to correspond to a narrower, longer ventricle, although the volume of the ventricular myocardium was unchanged. The muscular interventricular septum length was increased in *Robo1* mutants, despite the presence of ventricular septum defects (see Supplementary material online, *Figure S4E–G*). The reduced outflow tract rotation was even more pronounced in the *Robo1;Robo2* mutants (*Figure 3D*–*G*), possibly explaining the higher incidence of ventricular septum defects. These defects in outflow tract alignment are likely to underlie the ventricular septum defects in the absence of *Robo1* or both *Robo1* and *Robo2*.

### 3.3 A spectrum of valve malformations in Robo and Slit mutants

**Figure 4:**
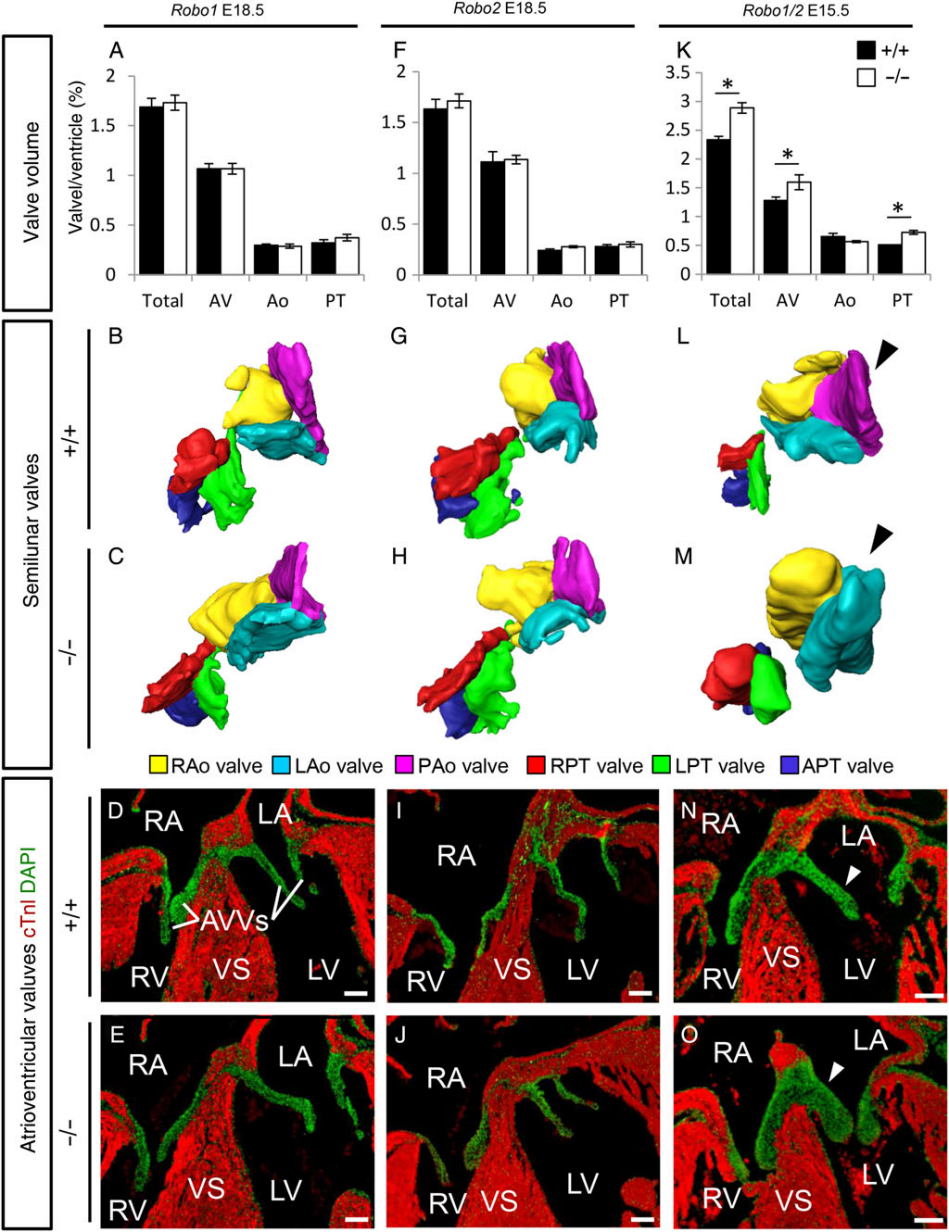
A spectrum of valve malformations in Robo mutants. (*A*–*O*) Analysis of the cardiac valves at the indicated developmental stages in *Robo1^+/+^* (*A*, *B,* and *D*) *Robo1^−/−^* (*A*, *C,* and *E*) *Robo2^+/+^* (*F*, *G,* and *I*) *Robo2^−/−^* (*F*, *H*, and *J*) *Robo1^+/+^;Robo2^+/+^* (*K*, *L,* and *N*), and *Robo1^−/−^;Robo2^−/−^* (*K*, *M,* and *O*) embryos. (*A*, *F,* and *K*) Measurements of the total valve (Total), atrioventricular valve (AV), aortic valve (Ao), and pulmonary trunk valve (PT) volume corrected for ventricular volume. (*A*) Total, *n* = 5, *P* = 0.47; AV, *P* = 0.60; Ao, *P* = 0.75; PT, *P* = 0.35. (*F*) Total, *n* = 5, *P* = 0.52; AV, *P* = 0.47; Ao, *P* = 0.08; PT, *P* = 0.92. (*K*) Total, WT *n* = 5, KO *n* = 3, *P* = 0.025; AV, *P* = 0.025; Ao, *P* = 0.30; PT, *P* = 0.025; Mann–Whitney *U* test. Note the overall increased valve volume in *Robo1^−/−^;Robo2^−/−^*. (*B*, *C*, *G*, *H*, *L*, and *M*) Example three-dimensional reconstructions as used for the volume measurements of the semilunar valves, seen from the ventricular side. Black arrowhead, note the absence of the posterior aortic valve in the *Robo1^−/−^;Robo2^−/−^* (M) embryo. (*D*, *E*, *I*, *J*, *N,* and *O*) Examples from immunohistochemistry sections (cTnI and DAPI) used for the measurements, showing the atrioventricular valves. White arrowheads, *Robo1^−/−^;Robo2^−/−^* (O) embryos show thickened valves. R/L/PAo, right/left/posterior aortic valves; R/L/APT, right/left/anterior pulmonary trunk valve; AVVs, atrioventricular valves. *P < 0.05. For other abbreviations, see the legend of *Figures 1* and *2*. Scale bars depict 100 µm.

**Figure 5:**
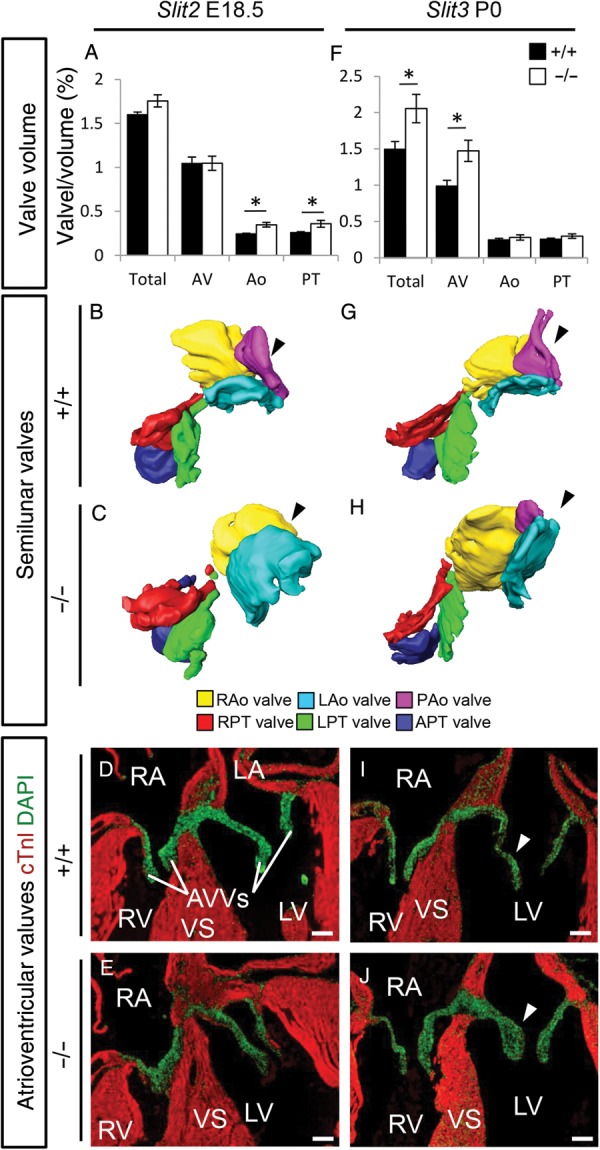
A spectrum of valve malformations in Slit mutants. (*A*–*J*) Analysis of the cardiac valves at the indicated developmental stages in *Slit2^+/+^* (*A*, *B,* and *D*), *Slit2^−/−^* (*A*, *C,* and *E*), *Slit3^+/+^* (*F*, *G,* and *I*), *Slit3^−/−^* (*F*, *H,* and *J*) embryos. (*A* and *F*) Measurements of the total valve (Total), atrioventricular valve (AV), aortic valve (Ao), and pulmonary trunk valve (PT) volume corrected for ventricular volume. (*A*) Total, *n* = 5, *P* = 0.08; AV, *P* = 0.92; Ao, *P* = 0.016; PT, *P* = 0.025. (*F*) Total, WT *n* = 5, KO *n* = 3, *P* = 0.025; AV, *P* = 0.025; Ao, *P* = 0.46; PT, *P* = 0.10; Mann–Whitney *U* test. Note that while there is overall increased valve volume in *Robo1^−/−^;Robo2^−/−^* mutants, *Slit2^−/−^* mutants only show increased semilunar (A) and *Slit3^−/−^* only increased total and atrioventricular valve volume (F). (*B*, *C*, *G,* and *H*) Three-dimensional reconstructions of the semilunar valves, seen from the ventricular side. Black arrowhead, note the absence of the posterior aortic valve in the *Slit2^−/−^* (*C*) while this valve is hypoplastic in the *Slit3^−/−^* (H) embryos. (*D*, *E*, *I,* and *J*) Immunohistochemistry sections (cTnI and DAPI) showing the atrioventricular valves. White arrowheads, *Slit3^−/−^* (*J*) embryos show thickened valves. R/L/PAo, right/left/posterior aortic valves; R/L/APT, right/left/anterior pulmonary trunk valve; AVVs, atrioventricular valves. *P < 0.05. For other abbreviations, see the legend of *Figure 1*. Scale bars depict 100 µm.

To investigate a role for defective cushion maturation in the formation of the ventricular septum defects, we next analysed the volume of the different cardiac cushion regions. We did not observe any differences in morphology or volume of the outflow tract or atrioventricular cushions in *Robo1*, *Robo2*, *Slit2*, or *Slit3* mutant embryos at E14.5 (data not shown). However, in all E12.5 *Robo1;Robo2* mutants, although cushion volume was not changed, the just forming aortic posterior cushion seemed absent (*Figure 3H* and *I*). In contrast, the pulmonary anterior cushion was forming normally. At E15.5, all *Robo1;Robo2* double mutants showed thickening of both the semilunar and atrioventricular valves, in combination with now clearly visible bicuspid aortic valves (*Figure 4K*–*O*; see Supplementary material online, *Figure S2E and F*). While the overall aortic valve volume was similar between *Robo1;Robo2* double mutants and wild-type littermates, this was caused by the absence of the posterior non-coronary aortic valve; the two remaining valves were thicker than their wild-type counterparts (*Figure 4K*–*M*). At E18.5, single *Robo1* or *Robo2* mutant valves remained indistinguishable from their wild-type littermates (*Figure 4A*–*J*). In contrast, E18.5 mice mutant for *Slit2* showed thickening of both the aortic and pulmonary semilunar valves, similar to the *Robo1;Robo2* double mutants; however, the atrioventricular valves retained a normal size (*Figure 5A*–*E*). As only one *Slit2* mutant recapitulated the bicuspid aortic valves as seen in the absence of both *Robo1* and *Robo2*, total aortic valve volume was significantly increased in mice lacking *Slit2* (*Figure 5A*–*C*). The thickness of the semilunar valves in *Slit3* mutants was relatively normal, but now the atrioventricular valves were strikingly thicker (*Figure 5F*–*J*). The posterior aortic semilunar leaflet, missing in the *Robo1;Robo2* double mutants, was highly hypoplastic in the absence of *Slit3*, but never completely absent (*Figure 5G* and *H*). These data indicate a role for Slit-Robo signalling during valve maturation and functional redundancy and requirement of *Robo1* and *Robo2* in both the outflow tract and the atrioventricular valve regions, whereas the Slit ligands show region-specific functions. Furthermore, these results underline the possible multi-causal origin of the ventricular septum defects.

### 3.4 Deregulation of *Robo2* and *Slit3* in the absence of the Robo1 receptor

**Figure 6:**
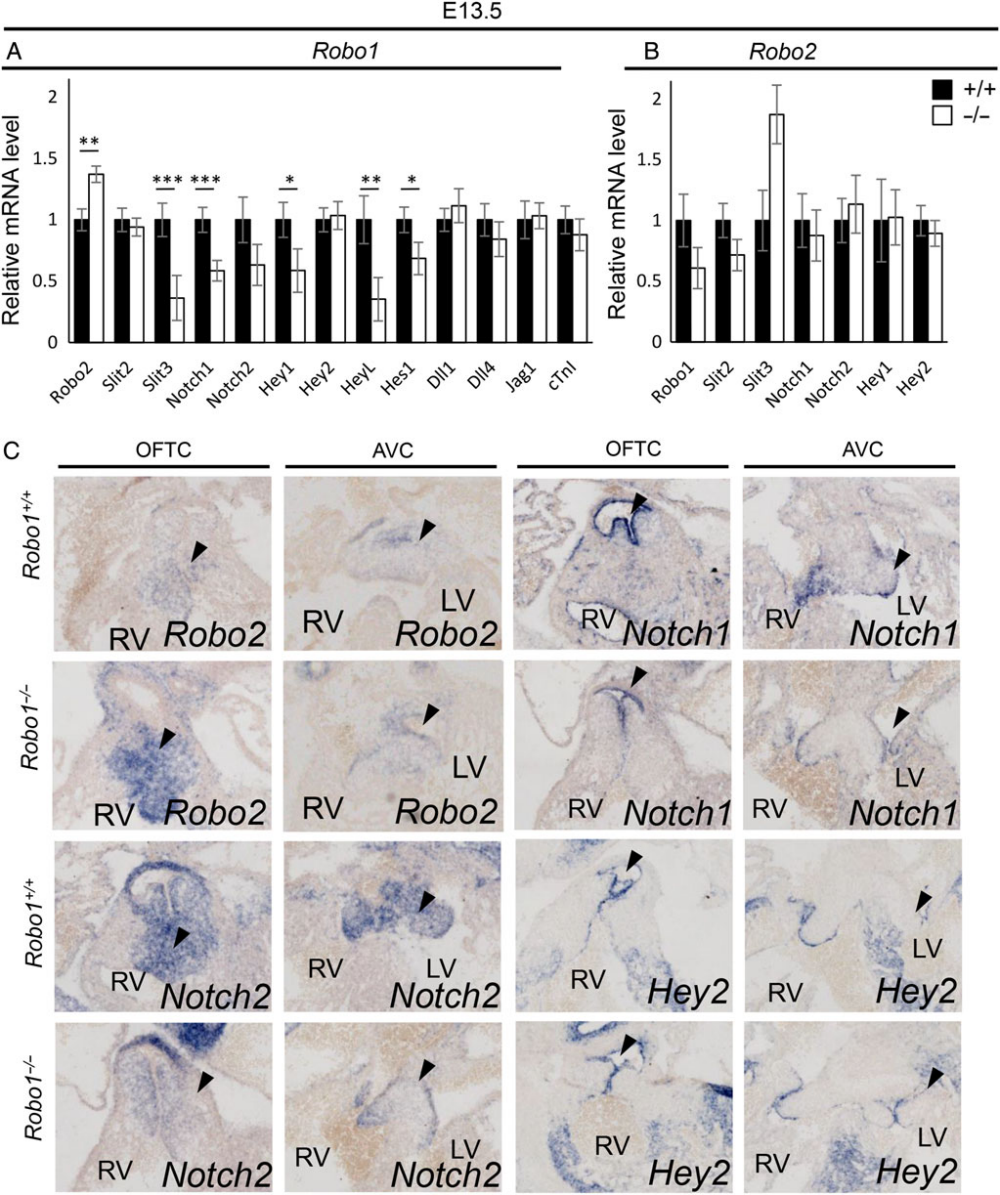
Down-regulation of Notch and downstream targets in the absence of *Robo1.* (*A* and *B*) Quantitative PCR on E13.5 *Robo1^+/+^* and *Robo1^−/−^* (*A*, *n* = 6) and *Robo2^+/+^* and *Robo2^−/−^* (*B*, *n* = 6) mRNA isolated from outflow tract with atrioventricular canal regions. All mutant levels visualized in relative levels to the wild-type expression set at 1. (*A*) Robo2, *P* = 0.007; Slit2, *P* = 0.62; Slit3, *P* ≤ 0.001; Notch1, *P* ≤ 0.001; Notch2, *P* = 0.09; Hey1, *P* = 0.026; Hey2, *P* = 0.82; HeyL, *P* = 0.003; Hes1, *P* = 0.029; Dll1, *P* = 0.53; Dll4, *P* = 0.39; Jag1, *P* = 0.86; cTnI, *P* = 0.45. (*B*) Robo1, *P* = 0.13; Slit2, *P* = 0.11; Slit3, *P* = 0.09; Notch1, *P* = 0.67; Notch2, *P* = 0.68; Hey1, *P* = 0.95; Hey2, *P* = 0.51; Student's *t*-test. (*C*) *In situ* hybridization on E13.5 *Robo1^+/+^* and *Robo1^−/−^* embryos for the indicated genes showing up-regulation of *Robo2*, down-regulation of *Notch1* and *Notch2,* but not *Hey2* in the absence of *Robo1*. Black arrow heads indicate the areas of differential expression in the cushions. *n* ≥ 3 embryos per gene. *P < 0.05, **P < 0.01 and ***P < 0.001. For abbreviations, see the legend of *Figure 1*. Scale bars depict 100 µm.

To address the possibility of functional redundancy and/or compensation of Slit/Robo genes in *Robo1^−/−^* mice, we isolated mRNA from E13.5 *Robo1* mutant and their wild-type littermate hearts of which the ventricles and atria were removed, leaving the regions containing the outflow tract with the atrioventricular canal. In the absence of *Robo1*, qPCR indicated an increase in *Robo2* expression levels. *Slit2* levels were normal, while the amount of Slit3 mRNA was greatly reduced (*Figure 6A*). Additionally, we confirmed the absence of *Slit1* and *Robo3* expression in the heart, while *Robo4* expression was unaltered (data not shown). These results were confirmed by *in situ* hybridization. While the reduction in *Slit3* expression was much less obvious with *in situ* hybridization than when assessed by qPCR (data not shown), Robo2 expression was visibly increased, particularly in the outflow tract cushions and forming membranous interventricular septum, where normally *Robo1* is highly expressed (*Figure 6C*; see Supplementary material online, *Figure S5A and B*). In the absence of *Robo2*, there was no significant change in Robo1 or Slit2 expression; in some, but not all mutants, Slit3 expression was much higher compared with the wild types (*Figure 6B*). These data suggest that most of the phenotype in *Robo1* mutants is rescued by the increase of *Robo2* expression and further suggests Slit3 as the main ligand for Robo1. While the increase in outflow tract *Robo2* expression seemed to rescue semilunar valve malformations in *Robo1* mutants, it failed to rescue the membranous septum defects or it may even underlie these defects.

### 3.5 Robo receptors are required for normal expression levels of the Notch signalling pathway

We have recently discovered the down-regulation of Notch2 and Hey1 during cortical development in *Robo1* mutants.^18^ Additionally, another group reported regulation of Hes1 by Robo2 in the same system.^19^ These observations, combined with the type of defects found in the *Slit* and *Robo* mutants, suggested Robo-Notch interaction in the heart as well.^3^ Thus, using qPCR, we tested the expression levels of a number of Notch and downstream target genes in E13.5 *Robo1* mutant and wild-type littermate mRNA isolated from the outflow tract and atrioventricular region. In agreement with our previous results in the developing cerebral cortex, *Robo1* mutant hearts showed decreased expression of downstream Notch targets *Hey1* and *Hes1*, although the decrease in *Notch2* did not reach significance. *Hes2* expression was below detection level in all samples (*Figure 6A*; data not shown). In addition, we found down-regulation of both *Notch1* and *HeyL*, suggesting broad regulation of the Notch signalling pathway (*Figure 6A*). The expression of Notch ligands *Dll1*, *Dll4*, and *Jag1* was unchanged, suggesting Robo does not regulate or influence Notch ligand presentation in interacting cells. The expression levels of *Notch1, Notch2, Hey1*, and *Hey2* were unaltered in *Robo2* mutants. The down-regulation of *Notch1*, and *Hey1,* but interestingly, most clearly *Notch2* in the absence of *Robo1* was confirmed by *in situ* hybridization (*Figure 6C*; see Supplementary material online, *Figure S5A–H*; data not shown); however, NICD1 expression was not visibly affected (see Supplementary material online, *Figure S6A and B*). Interestingly, *Hey2* expression was unaltered in *Robo1* mutants, both by qPCR and *in situ* hybridization (*Figure 6A*–*C*; see Supplementary material online, *Figure S5G and H*).

**Figure 7:**
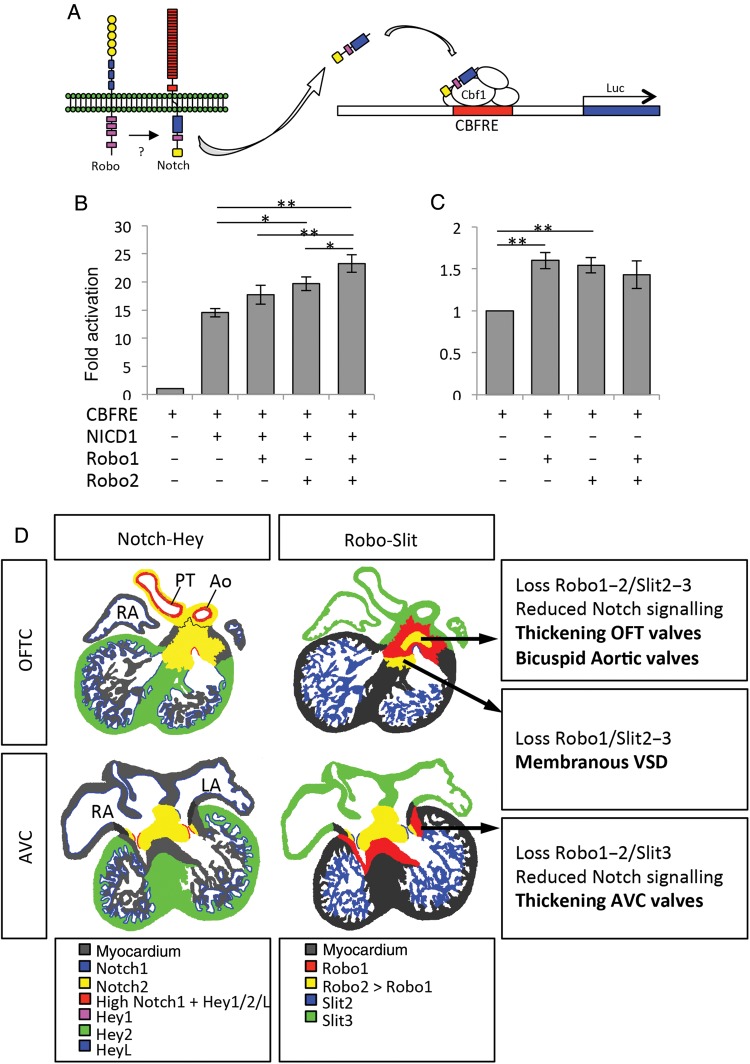
Robo activates CBFRE-luciferase activity. (*A*) Robo and Notch are both membrane-bound receptors with intracellular signalling domains. Cleavage and binding of the intracellular domain of Notch to Cbf1 activates the CBFRE-luciferase reporter construct. (*B* and *C*) CBFRE-luciferase activation in the presence of the indicated transfected genes in COS7 cells. (*B*) *P* = 0.65, *P* = 0.045, *P* = 0.01 compared with NICD1 alone, respectively. Robo1 and 2 with NICD1 compared with Robo1 with NICD1 *P* = 0.007, to Robo2 with NICD1 *P* = 0.041, *n* = 5. (*C*) *P* = 0.004, *P* = 0.004, *P* = 0.14 compared with NICD1 alone, respectively, *n* = 5; one-way ANOVA with Bonferroni's multiple comparison test. (*D*) Scheme showing the similarity of the expression patterns between the Slit-Robo and Notch-Hey genes, with an overview of the observed defects. Luc, luciferase. *P < 0.05, **P < 0.01. For other abbreviations, see the legend of *Figure 1*.

Next, we tested whether Robo receptors might directly enhance Notch signalling *in vitro*. Thus, we performed luciferase assays on COS7 cells, which were co-transfected with a plasmid containing four Notch-responsive Cbf1-binding elements and the basal simian virus 40 (SV40) promoter upstream of a luciferase cassette (CBFRE-luc), a plasmid with the Notch1 intracellular domain (NICD1) and/or Robo receptor expression plasmids (*Figure 7A*–*C*). In the control experiments, the presence of NICD1 expression strongly stimulated Notch-responsive CBFRE-luciferase activity (*Figure 7B*). This confirmed that the Notch-responsive element is activated by NICD1, with a 14-fold increase over basal expression levels found with an identical plasmid lacking NICD1 (*Figure 7B*). Co-transfection of Robo1 or Robo2-expressing plasmid and NICD1 further increased luciferase activation (respectively 18- and 20-fold), albeit Robo1 never reached significant levels. These results indicate that Robo2 is more important for regulating Notch signalling than Robo1. However, co-transfection of both Robo1 and Robo2 with NICD1 resulted in further increase in luciferase activity by 23-fold. This suggests that Notch signalling in the heart is spatially regulated by Robo expression, with maximum regulation when both Robo1 and Robo2 are present. Both Robo1 and Robo2 also minimally activated CBFRE-luciferase activity in the absence of NICD1 (1.6-, 1.5-, or 1.4-fold, respectively, *Figure 7C*). This might be caused by low levels of endogenous NICD1 present in COS7 cells or by direct regulation of Cbf1-responsive elements by Robo receptors. However, our data indicate that Robo receptors mainly function synergistically with NICD1, possibly by stabilizing N1ICD in the nucleus, although the nature of interaction, be it direct or indirect, will need to be further examined. Combined with the qPCR data, these results show that Robo1 and Robo2 receptors are capable of activating Notch signalling, suggesting that reduced activation of the Notch signalling pathway might underlie the defects observed in the mutants for Slit or Robo.

## 4. Discussion

### 4.1 The Slit-Robo pathway as a new player in cardiac cushion development

Slit-Robo signalling has been implicated in heart tube assembly and lumen formation in *Drosophila* and zebrafish development,^10–13^ and in cardiac chamber formation,^14^ pericardial development,^16^ and cardiac neural crest migration and adhesion during murine heart development.^15,16^ However, a role in cardiac cushion development has not yet been described. We have shown here how genes of the Slit-Robo signalling pathway locally regulate membranous ventricular septum and valve formation. Both Robo1 and Robo2 receptors and their ligands Slit2 and Slit3 were expressed in or surrounding the cushions, and later valves, throughout development. The Robo1 receptor seemed the most crucial during heart development, which may be caused by the fact that Robo1, but not Robo2, can interact with Neuropilin1, a receptor required for outflow tract cushion development and membranous ventricular septum formation.^20,21^ Additionally, at least part of the difference between the phenotype in the *Robo1* and *Robo1;Robo2* mutants might be explained by rescue of *Notch* levels by an increase in *Robo2* expression in the *Robo1* mutant.

Bicuspid aortic valves were present in all *Robo1;Robo2* double mutants analysed and were recapitulated, albeit with lower penetrance, in the absence of *Slit2* and hypoplastic in the absence of *Slit3*, suggesting the requirement of all four genes for proper formation of the aortic semilunar valves. Thickening of the atrioventricular valves was only observed in *Robo1;Robo2* and *Slit3* mutants. This is interesting, as Slit2 is expressed in both locations in the endocardium covering the valves, whereas Slit3 is expressed in the neighbouring myocardium. This raises the question as to the role of Slit2 in this region but, most importantly, how does Slit3 reach the atrioventricular cushions as it is expressed in the atrial and outflow tract myocardium, but not in or directly surrounding the cushions? The expression level of *Slit3* might be below detection level with *in situ* hybridization; however, Slit ligands have also been described able to act over long distances,^22^ indicating Slit3 might be able to reach the cushions from the relatively close by myocardium. Furthermore, some of the observed defects might be secondary to other abnormalities and not primary defects, possibilities that can only be investigated using tissue-specific conditional mutants. Taken together, our data demonstrate the local tight control of valve and membranous ventricular septum formation by the selective spatial interaction of genes of the Robo signalling pathway.

### 4.2 Defective Slit3-Robo1 signalling results in membranous ventricular septum defects

Recently, we have shown a role for Slit-Robo signalling in the development of both the systemic venous return to the heart as well as the pericardium. These pericardial defects seemed to be caused by abnormal localization of the caval veins combined with ectopic pericardial cavity formation, brought about by increased cell death and impaired adhesion and migratory responsiveness of the cardiac neural crest.^16^ In contrast, neural crest cell contribution to the outflow tract cushions was normal in *Robo1* mutants. However, as the area of impaired outflow tract cushion closure in *Robo1/2* double mutants was located in the neural crest derived part of the cushion, this still indicates a possible other role for the neural crest in the development of the membranous septum defects. More importantly, the expression of *Robo1* as well as *Slit2* and *3* in the second heart field suggests that disruptions in second heart field contribution to the outflow tract are another likely cause of the observed defects. The exact role of Slit-Robo signalling in the second heart field will still need to be further examined. Defects in both the second heart field and cardiac neural crest are linked to malrotation of the aorta and pulmonary trunk at their connection to the ventricle,^4,5^ as found to some degree in the *Robo1* and *Robo1/2* mutants, which is a known cause of membranous ventricular septum defects.

In addition to the septum defects, the neural crest has been linked with providing instructive signals for remodelling of the semilunar valves. Correct signalling and tissue–tissue interactions among second heart field, neural crest, and endocardial cushion mesenchyme are required for normal valve formation.^23^ The expression patterns of Robo1 and Slit3 in the second heart field and neural crest, Robo1 and Robo2 in the cushions, and Slit2 in the endocardium also suggest signalling between these cell populations.

### 4.3 Several downstream effectors of Notch are affected by disrupted Slit-Robo signalling

Notch intercellular signalling has been implicated in multiple aspects of heart development including cushion development, trabecular growth, atrioventricular patterning, neural crest differentiation, and outflow tract development.^3^ The Notch signalling pathway is known to regulate both the endocardial-to-mesenchymal transformation underlying early cushion development and their subsequent remodelling into the thin mature leaflets of the atrioventricular and semilunar valves.^3,24^

Bicuspid aortic valves are among the most common of congenital defects affecting 1–2% of the population^25,26^ and are associated with ventricular septum defects and arterial malformations such as aortic coarctation, aneurisms of the descending aorta, and carotid and vertebral artery defects. Bicuspid aortic valves have only two complete leaflets, while the third leaflet is either absent or incomplete.^26^ To date, NOTCH1 is the only transcriptional regulator linked to bicuspid aortic valve disease in humans in a limited number of familial cases and ∼4% of sporadic cases.^27–29^ While mice ubiquitously mutant for Notch1 die early during development due to severe defects, tissue-specific Notch inhibition has shown to mimic the defects seen in humans with mutations in NOTCH1.^30^ The defects observed in Slit-Robo mutants, in which Notch signalling is down-regulated, also closely resemble defects caused by human NOTCH1 mutations.

Notch signalling exerts its function through activation of effector genes such as the Hey and Hes families. The observed decrease of *Notch1* and *Notch2* in *Robo1* mutants subsequently resulted in down-regulation of the downstream targets *Hey1*, *HeyL*, and *Hes1*, although not *Hey2*. Combined loss of *Hey1* and *HeyL* results in membranous ventricular septum defects and dysplastic atrioventricular and pulmonary valves, similar to the defects observed in the absence of Slit-Robo signalling.^31^ *Hey2* expression was unaltered in *Robo1* mutants, in line with previous findings which suggested that aberrations in Notch signalling do not always result in changes in *Hey2* expression.^32^ Consequently, the cardiac defects reported in *Hey2* mutants are largely different from those seen in *Robo1;Robo2* animals.^33^ The dextra-posed aorta phenotype present in Hes1 mutants was partially recapitulated in Robo1 mutants, consistent with the fact that Hes1 is down-regulated, but not absent in these mice.^34,35^ The extensive range of pharyngeal arch artery defects in the absence of Hes1 warrants further detailed investigation in *Slit* and *Robo* mutants in future studies.

As both Robo and Notch are transmembrane receptors, the question is how Robo regulates Notch signalling. There can be direct interaction at the membrane level, interaction of the intracellular domains either at the membrane or in the nucleus, or the intracellular domain of Robo independently binds to Cbf1 or Notch-responsive elements. Modulation of transcription by Robo signalling has been suggested in other systems, although no direct targets were identified.^36,37^ Transcriptional regulation by the intracellular domain of Robo is similar to a mechanism described in a recent study on the regulation of Hes1 by Robo2 during cortical development.^19^ In contrast to the latter study, however, we found that Notch expression is down-regulated in the absence of Robo, suggesting that Robo might activate Notch signalling instead of directly regulating Notch-responsive elements independently. The fact that the luciferase experiments suggest a more important role for Robo2 than Robo1 in the regulation of Notch signalling, in contrast to the mutant data, also needs further attention. This difference might be caused by the *in vitro* conditions, for example by a different response in COS7 cells, the lack of Slit ligands *in vitro*, or point to the involvement of other factors. The luciferase experiments do confirm maximal regulation in the presence of both receptors as found in the mutant analysis. An interesting observation is the lack of requirement for Slit ligand in the luciferase experiments, whereas the Slit2 and Slit3 mutant data indicate the requirement of Slit-Robo binding. This might be caused by the strong overexpression of Robo receptors in these experiments. The genes of the Slit-Robo and Notch-Hey/Hes pathways show strikingly similar expression patterns in the developing heart (*Figures 1*, *6*, and *7D*).^3^ Notch1 overlaps with the expression of Slit2 and Hey genes in the endothelium surrounding the cushions, while Notch2 is expressed in the cushions overlapping with Robo1 and Robo2. Hey2 and Slit3 show complementary gene expression in ventricle and atrium with outflow tract myocardium, respectively (*Figure 7D*). These similar patterns with different combinations of Notch-Hey and Slit-Robo genes in different cell types might explain the observed difference in phenotype between *Slit* and *Robo* mutants. The identification of the exact cellular interactions and signals required for the modulation of Notch by the Slit-Robo signalling pathway will be the focus of future studies.

Here, we uncovered a novel signalling pathway controlling membranous ventricular septum and cardiac valve formation, the Slit-Robo pathway. Further study of the identified range of valve defects, and in particular the already early in development recognizable bicuspid aortic valves, might help understand the aetiology of common congenital valve defects found in patients.

## Supplementary material

Supplementary material is available at *Cardiovascular Research* online.

## Funding

This work was supported by the Wellcome Trust (Programme Grant 089775) and the British Heart Foundation (PG/12/39/29626 to M.T.M.M.). M.T.M.M. was a recipient of an EMBO Long-Term Fellowship grant (ALTF 441-2010) and Netherlands Organisation for Scientific Research Rubicon grant (825.10.025). Funding to pay the Open Access publication charges for this article was provided by the Welcome Trust.
